# In patients with combined clavicle and multiple rib fractures, does fracture fixation of the clavicle improve clinical outcomes? A multicenter prospective cohort study of 232 patients

**DOI:** 10.1097/TA.0000000000004001

**Published:** 2023-05-11

**Authors:** Ruben J. Hoepelman, Rens A. van der Linde, Frank J.P. Beeres, Reinier B. Beks, Arthur A.R. Sweet, Koen W.W. Lansink, Bas van Wageningen, Tjarda N. Tromp, Fabrizio Minervini, Björn-Christian Link, Nicole M. van Veelen, Jochem M. Hoogendoorn, Mirjam B. de Jong, Mark C.P. van Baal, Luke P.H. Leenen, Rolf H.H. Groenwold, Roderick M. Houwert, Frank F. IJpma

**Affiliations:** From the Department of Trauma Surgery (R.J.H., R.B.B., A.A.R.S., M.B.d.J., M.C.P.v.B., L.P.H.L., R.M.H.), University Medical Center Utrecht, Utrecht; Department of Trauma Surgery (R.A.v.d.L., F.F.I.), University Medical Center Groningen, University of Groningen, Groningen, the Netherlands; Department of Orthopedic and Trauma Surgery (F.J.P.B., B.-C.L., N.M.v.V.), Luzerner Kantonsspital, Lucerne, Switzerland; Department of Trauma Surgery (K.W.W.L.), Elisabeth-TweeSteden hospital, Tilburg; Department of Trauma Surgery (B.v.W., T.N.T.), Radboud University Medical Center, Nijmegen, the Netherlands; Department of Thoracic Surgery (F.M.), Luzerner Kantonsspital, Lucerne, Switzerland; Department of Trauma Surgery (J.M.H.), Haaglanden Medical Center, the Hague; and Department of Clinical Epidemiology (R.H.H.G.)and Department of Biomedical Data Sciences (R.H.H.G.), Leiden University Medical Center, Leiden, the Netherlands.

**Keywords:** Rib fracture, clavicle fracture, combined clavicle and rib fractures, costoclavicular fractures

## Abstract

Clavicle and rib fractures are often sustained concomitantly. The combination of injuries may result in decreased stability of the chest wall, making these patients prone to (respiratory) complications and prolonged hospitalization. This study aimed to assess whether adding chest wall stability by performing clavicle fixation improves clinical outcomes in patients with concurrent clavicle and rib fractures.

**METHODS:**

A prospective multicenter study was performed including all adult patients admitted between January 2018 and March 2021 with concurrent ipsilateral clavicle and rib fractures. Patients treated operatively versus nonoperatively for their clavicle fracture were matched using propensity score matching. The primary outcome was hospital length of stay (HLOS). Secondary outcomes were intensive care unit length of stay, duration of mechanical ventilation, pain, complications, and quality of life at 6 weeks and 12 months of follow-up.

**RESULTS:**

In total, 232 patients with concomitant ipsilateral clavicle and rib fractures were included. Fifty-two patients (22%) underwent operative treatment of which 39 could be adequately matched to 39 nonoperatively treated patients. No association was observed between clavicle plate fixation and HLOS (mean difference, 2.3 days; 95% confidence interval, −2.1 to 6.8; *p* = 0.301) or any secondary endpoint. Eight of the 180 nonoperatively treated patients (4%) had a symptomatic nonunion, for which 5 underwent secondary clavicle fixation.

**CONCLUSION:**

We found no evidence that, in patients with combined clavicle and multiple rib fractures, plate fixation of the clavicle reduces HLOS, pain, or (pulmonary) complications, nor that it improves quality of life.

**STUDY TYPE:**

Therapeutic/Care Management; Level III.

## Abstract 2

Rib fractures and clavicle fractures frequently occur concurrently, however, there is no literature available on this patient group. This study provides the largest prospective cohort on this combined injury which can guide surgeons in their clinical decision making

## Graphical abstract

**Figure:**
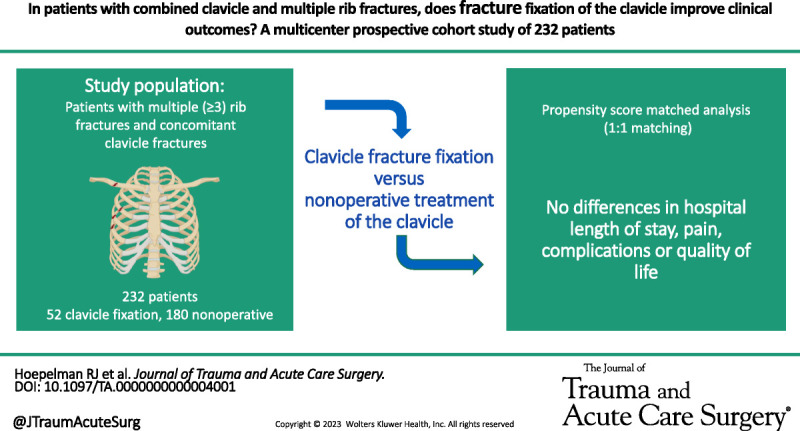

Thoracic injuries are common, occurring as both isolated chest injuries and multiple injuries in patients.^1,2^ In multiple injury patients who sustained blunt chest trauma, the most prevalent fractures include rib (86%), clavicle fractures (19%), or a combination of these injuries (19%).^3,4^ Both injuries are considered indicators of the severity of thoracic trauma and may independently increase the risk of mortality.^5–9^ The combination of multiple rib and clavicle fractures could result in decreased stability, compliance, and respiratory support of the chest wall. Therefore, patients with these combined injuries after blunt thorax trauma seem prone to (respiratory) complications and longer hospitalization.^10,11^ There is an ongoing debate whether fixation of the clavicle fracture in patients with multiple concomitant rib fractures could be beneficial to provide additional stability to the chest wall and thereby improve clinical outcomes.^4^

Despite the vast amount of literature describing rib fractures and clavicle fractures as individual entities, the mutual impact and subsequent management of these combined injuries remain unclear. So far, only three studies reported on treatment outcomes in patients with both clavicle and rib fractures.^12,13^ Graf et al.^13^ reported on a chart review of 60 patients with blunt chest trauma and concomitant clavicle fractures, and they did not find any differences between early clavicle fixation versus nonoperative treatment of clavicle fractures. Langenbach et al.^10^ reported on 11 patients with flail chest injuries and clavicle fractures, all of whom underwent operative treatment for both fractures and recovered uneventfully. Solberg et al.^12^ presented a retrospective case series of blunt chest trauma patients with combined clavicle and multiple rib fractures, comparing operative (n = 9) versus nonoperative (n = 7) treatment of the rib fractures. However, no definitive conclusion could be drawn from these results because of the small number of included patients. In conclusion, the available literature is insufficient to determine whether clavicle fixation improves outcomes in patients with concomitant clavicle and rib fractures.

We, therefore, performed this study with the following research question: does clavicle fixation in patients with combined rib and clavicle fractures affect hospital length of stay (HLOS), quality of life, and other in-hospital outcomes?

## PATIENTS AND METHODS

### Study Design

Data for the current study on combined multiple rib and clavicle fractures were gathered from the database of the OPVENT study. The OPVENT study is an international multicenter prospective cohort study consisting of 1,014 patients with multiple rib fractures and/or flail chest and compared nonoperative treatment with rib fixation. This study was performed in six level 1 trauma centers between January 2018 and March 2021. The study protocol and results on operative versus nonoperative treatment of multiple rib fractures have been published.^13,14^

In the current study, we focus solely on patients with rib fractures in combination with a clavicle fracture. All consecutive patients of 18 years and older with computed tomography confirmed that multiple rib fractures (defined as three or more ipsilateral rib fractures) combined with an ipsilateral clavicle fracture were eligible for inclusion. The exclusion criteria were cognitive impairment, nontraumatic rib fractures, and rib fractures due to cardiopulmonary resuscitation. This study adhered to the Strengthening the Reporting of Observational Studies in Epidemiology guidelines (Supplemental Digital Content, Supplementary Data 1, http://links.lww.com/TA/D10).^15,16^

### Study Population

Patient characteristics measured at hospital admission included age, sex, body mass index (BMI), American Society of Anesthesia score, presence of chronic obstructive pulmonary disease, smoking status, trauma mechanism, Abbreviated Injury Scale (AIS) score, Injury Severity Score (ISS), number of fractured ribs, clinical or radiological flail chest (defined as three or more sequential rib fractures in two or more places with [clinical] or without [radiological] paradoxical movement of the chest wall), concomitant injuries (i.e., pulmonary contusion, pneumothorax, hemothorax, and sternum fracture), and laboratory results (specifically pH and base excess).

### Treatment

Conservative treatment of clavicle fractures consisted of a sling for 1 week after which shoulder exercises commenced. Fracture displacement of ≥1 shaft width and/or shortening of ≥1 cm were indications for recommending operative treatment.^17^ The decision for operative treatment was made within <72 hours of admission. Operative treatment of the clavicle was performed with open reduction and internal fixation using plate osteosynthesis. Some patients with displaced clavicle fractures might not have had surgery because of shared decision making or differences in surgeon preference with regard to operative treatment in some centers. Three of the study hospitals had a preference for operative treatment (Luzerner Kantonsspital, 24 of 31 [77%]; Elisabeth Tweesteden Ziekenhuis, 4 of 12 [33%]; and University Medical Center Groningen, 9 of 37 [24%]), whereas the other three hospitals generally treated patients conservatively (University Medical Center Utrecht, 10 of 98 [11%]; Radboud University Medical Center, 2 of 18 [11%]; Haaglanden Medical Center, 3 of 27 [11%]). Some patients with combined injuries underwent rib fracture fixation as well. In case of operative treatment of both the clavicle and ribs, these procedures were performed in the same session. The indication for rib fixation was based on clinical and radiological assessment by a trauma surgeon. Indications for rib fixation included flail chest, severely displaced fractures, and chest wall deformity, as well as failure to wean from mechanical ventilation or uncontrolled persistent pain despite maximum administration of analgesia. All rib fractures were treated using locking plates (MatrixRib; Depuy Synthes®, Amersfoort, The Netherlands). The University Medical Center Utrecht, Luzerner Kantonsspital, and Elisabeth Tweesteden Ziekenhuis performed rib fixation according to protocol in patients with previously mentioned indications. The University Medical Center Groningen, Radboud University Medical Center, and Haaglanden Medical Center did not perform any rib fixations.

### Primary and Secondary Outcomes

Our primary outcome was to assess whether fixation of a clavicle fracture in patients with combined rib and clavicle fractures affects HLOS. Secondary outcomes included intensive care unit length of stay, duration of mechanical ventilation (DMV), need for tracheostomy, pneumonia rate and other in-hospital complications, in-hospital mortality rate, and general pain at days 3, 5 and 7 (measured by nurses using a numeric rating scale [NRS]). Midterm and long-term outcomes were measured at the outpatient clinic visit at 6 weeks and using telephone interviews after 12 months. These measures included pain when breathing and coughing (measured using the NRS), quality of life (measured using the EQ5D-5L), dyspnea burden (measured using the modified Medical Research Council dyspnea scale), and return to work and sports in weeks. Other complications included fracture-related infection (defined by the fracture-related infection^18^ and symptomatic clavicle nonunion). Pneumonia was defined as clinical signs and symptoms (two or more present: temperature >38.5°C, auscultation with suspicion for infiltrate or purulent sputum) and/or additional tests (thoracic radiographs with signs of infiltrate, leukocytosis, elevated C-reactive protein) requiring antimicrobial therapy. Acute respiratory distress syndrome was defined according to the Berlin definition.^19^ Symptomatic nonunion was defined as the presence of unsuccessfully healed ribs, confirmed by computed tomography scan, at least 6 months after trauma, with clinical evidence of pain. The EQ5D-5L is a patient-reported validated questionnaire that measures health-related quality of life based on five dimensions of health: mobility, self-care, usual activities, pain/discomfort, and anxiety/depression. The EQ5D score ranges from 0 to 1, with higher scores indicating better quality of life. In addition, it contains a visual analog scale of 0 to 100 where 0 is the worst health imaginable and 100 is the best health imaginable for the patient. The modified Medical Research Council is a five-category scale that characterizes the level of dyspnea with physical activity, where 0 indicates dyspnea only with strenuous exercise and 4 means already being dyspneic when getting dressed.

### Ethical Approval

The study was registered in the Netherlands Trial Registry (NTR6833). The institutional review boards of all participating centers approved the study protocol. Informed consent was obtained from all participants

### Sample Size and Statistical Analyses

All analyses were performed using R statistical software v4.1.2.22 (The R Foundation for Statistical Computing, Vienna, Austria). Data were presented as mean with SD, median with interquartile range, or frequencies and percentages in case of nominal data. The differences in the distribution of the data between study groups were quantified using standardized mean differences (SMDs). We performed multiple imputation, creating 25 imputed dataset, to impute missing values for baseline characteristics: AIS head/face/thorax/extremities (4% [39 of 927]), base excess (29% [266 of 927]), BMI (3% [25 of 927]), ISS (4% [39 of 927]), pH (29% [265 of 927]), and smoking status (2% [16 of 927]), using the “mice” algorithm in R. Within each data set, we performed propensity score (PS) matching to control for confounding. The PS was estimated using binary logistic regression analysis, with clavicle fixation as the dependent variable and age, sex, smoking status, chronic obstructive pulmonary disease, BMI, American Society of Anesthesia score, trauma mechanism, AIS head/face/thorax/abdomen/extremities, ISS, number of fractured ribs, clinical/radiological flail chest, rib fixation, and concomitant injuries as covariates in the model. We chose 1:1 nearest neighbor matching, with a caliper of 0.2 of the SD of the natural logarithm of the PS using the MatchIt algorithm in R. After PS matching, the baseline characteristics of the two groups were compared and quantified using the SMDs, where an SMD of <0.1 indicates adequate comparability between groups. The primary analyses were conducted with the matched cohort. The relationship between clavicle fixation and outcomes was assessed using linear regression analysis for continuous outcomes and binary logistic regression analysis for binary outcomes. Secondary, we performed a multiple regression analysis of all subjects included in the study, with correction for confounding by including potential confounders as covariates in the regression model. A sensitivity analysis was performed on the HLOS, which was measured in this analysis from operation to discharge. Follow-up for the nonoperative group started 4.5 days after admission, which was the mean time to surgery. This is known as the landmark method to correct for possible immortal time bias.^20^ Analyses were performed separately for each imputed data set, and the results were pooled using Rubin's rules.

## RESULTS

### Patient Characteristics

**Figure 1:**
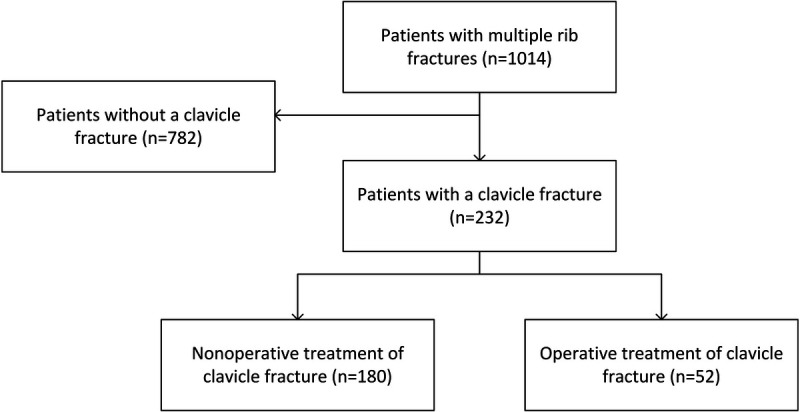
Flowchart of included patients.

From a total of 1,014 patients, 232 had concomitant clavicle and rib fractures of whom 52 patients (22%) underwent operative treatment for the clavicle fractures and 180 patients (78%) received nonoperative treatment (Fig. 1). Follow-up was completed in March 2022 with a completion rate of 85%. The mean ± SD time from admission to surgery of the clavicle was 4.5 ± 4.3 days. After PS matching, on average, 39 patients from the operative group were adequately matched to 39 patients from the nonoperative group. All baseline demographic variables before and after PS matching are available in Table 1.

**TABLE 1:** Baseline Characteristics of Patients With Combined Clavicle and Rib Fracture, Before and After PS Matching

	Before Matching			After Matching		
Variable	Nonoperative (n = 180)	Clavicula Fixation (n = 52)	SMD	Nonoperative (n = 39)*	Clavicula Fixation (n = 39)*	SMD**
Age (mean ± SD), y	59.6 ± 15.2	50.2 ± 14.9	0.623	51.5 ± 14.3	51.4 ± 15.1	0.009
Male, n (%)	138 (76.7)	44 (84.6)	0.202	30 (78)	32 (81)	0.070
ASA score, n (%)			0.541			0.044
1–2	142 (78.9)	50 (96.2)		37 (95)	37 (95)	
>2	38 (21.1)	2 (3.8)		2 (5)	2 (5)	
BMI (mean ± SD), kg/m^2^	25.5 ± 3.8	25.9 ± 4.5	0.094	25.4 ± 3.8	25.4 ± 4.1	0.016
COPD, n (%)	9 (5)	0 (0)	0.324	0 (0)	0 (0)	<0.001
Current smoker, n (%)	32 (18)	4 (8)	0.306	4 (11)	4 (11)	0.010
Trauma mechanism, n (%)			0.475			0.049
Motor vehicle accident	118 (65.6)	44 (84.6)		32 (83)	31 (82)	
Fall from height/stairs	54 (30)	6 (11.5)		6 (15)	6 (15)	
Other	8 (4)	2 (4)		1 (2)	2 (3)	
ISS (mean ± SD)	21.1 ± 9.3	19.2 ± 7.5	0.222	19.1 ± 9.5	19.0 ± 7.1	0.012
TTSS (mean ± SD)	9.3 ± 3.5	7.8 ± 3.4	0.447	8.3 ± 3.2	8.2 ± 3.3	0.012
AIS, median (IQR)						
Head	1 (0–3)	0 (0–1)	0.481	0 (0–1)	0 (0–2)	0.020
Face	0 (0–1)	0 (0–.75)	0.002	0 (0–1)	0 (0–1)	0.011
Thorax	3 (3–3)	3 (3–3)	0.067	3 (3–3)	3 (3–3)	0.007
Abdomen	0 (0–0)	0 (0–0)	0.012	0 (0–0)	0 (0–0)	0.007
Extremities	2 (2–2)	2 (2–2)	0.271	2 (2–2)	2 (2–2)	0.005
No. rib fractures, median (IQR)	7 (5–9)	7 (4–8)	0.158	6 (4–8)	6 (4–8)	0.003
Radiological flail chest, n (%)	52 (29)	16 (31)	0.041	10 (27)	10 (27)	0.007
Clinical flail chest, n (%)	18 (10)	3 (6)	0.157	2 (5)	2 (5)	0.010
Bilateral rib fractures, n (%)	44 (24)	10 (19)	0.126	8 (21)	8 (21)	0.003
Rib fracture location						0.098
High (ribs 1–4)	169 (93.9)	51 (98.1)	0.398	35 (89.9)	36.9 (94.6)	
Middle (ribs 5–8)	171 (95)	47 (90.4)	0.368	35.6 (91.2)	33.9 (86.8)	
Low (ribs 9–12)	74 (41.1)	21 (40.4)	0.999	13.5 (34.6)	16.5 (42.3)	
Clavicle fracture location						0.040
Medial	21 (12.2)	4 (8.3)		4.9 (13.4)	5.2 (14.3)	
Midshaft	106 (61.6)	33 (68.8)		22.6 (61.8)	22.7 (62.2)	
Lateral	45 (26.6)	11 (22.9)		9.1 (24.9)	8.6 (23.4)	
Clavicle dislocation						0.048
Not dislocated	60 (34.9)	6 (12.5)		5.6 (15.4)	6 (16.4)	
<1 Shaft width dislocation	43 (25)	17 (35.4)		13 (35.5)	12.2 (33.3)	
≥1 Shaft width dislocation	69 (26.2)	25 (52.1)		17.9 (49.1)	18.4 (50.3)	
Concomitant thoracic injuries, n (%)						
Pulmonary contusion, n (%)	91 (51)	20 (39)	0.245	17 (44)	17 (44)	0.010
Pneumothorax, n (%)	113 (63)	28 (54)	0.182	22 (56)	23 (58)	0.050
Hemothorax, n (%)	44 (24)	15 (29)	0.100	12 (32)	12 (32)	0.013
Sternum fracture, n (%)	17 (9)	1 (2)	0.329	1 (4)	1 (4)	0.054
Blood pH (mean ± SD)	7.36 ± 0.09	7.36 ± 0.05	0.066	7.36 ± 0.12	7.36 ± 0.10	0.024
Base excess (mean ± SD)	−0.6 ± 3.76	−0.83 ± 3.66	0.060	−0.98 ± 4.4	−1.17 ± 4.2	0.044
Rib fixation, n (%)	22 (12.2)	11 (21.2)	0.241	5 (13)	5 (13.0)	0.018

*Numbers indicate the average of 25 matched imputed sets.

**SMD <0.1 indicates adequate matching.

ASA, American Society of Anesthesiologists; COPD, chronic obstructive pulmonary disease; IQR, interquartile range; MVA, motor vehicle accident; TTSS, Thoracic Trauma Severity Score.

### Primary Outcome

Median HLOS was 6 (3–13) days for the nonoperative group and 9 (5–17) days for the operative group (Table 2). After PS matching, no association was found between clavicle fixation and HLOS (mean difference, 2.3 days; 95% confidence interval [CI], −2.1 to 6.8; *p* = 0.304). Adjustment for confounding through multiple regression analysis yielded similar results Supplemental Digital Content, Supplementary Table 1, http://links.lww.com/TA/D11).

**TABLE 2:** In-hospital Outcomes and Complications After PS Matching of Patients With Combined Clavicle and Rib Fractures

Outcome Variable	Multiple Rib Fractures and Clavicle Fracture					
Median (IQR) or n (%)	Nonoperative (n = 39*)	Clavicle Fixation (n = 39*)	Regression Coefficient (*b*)	95% CI	SE	*p*
HLOS	6 (3–13)	9 (5–17)	2.3	−2.1 to 6.8	2.280	0.304
HLOS from clavicle fixation	3 (0–9.25)	5 (2–10)	2.4	−2.0 to 6.8	2.251	0.286
ICU length of stay	3 (1–12)	2 (1–4)	−0.2	−2.1 to 1.7	0.967	0.801
Duration of invasive mechanical ventilation	3 (1–13)	7 (2–8)	−0.6	−2.1 to 0.8	0.763	0.380
Duration of epidural analgesia	5 (4–6)	6 (5–9)	1.5	−0.03 to 2.9	0.748	0.054
Duration of intravenous analgesia	2.5 (1–5.75)	6 (5–9)	0.9	−1.4 to 3.1	0.857	0.452
NRS (pain)						
Day 3	3 (2–4)	2 (1–3)	−0.2	−1.1 to 0.7	0.451	0.614
Day 5	2 (2–3.5)	2 (1–3)	−0.1	−0.9 to 0.7	0.419	0.780
Day 7	2 (2–3)	2 (1–3)	0.1	−0.5 to 0.8	0.327	0.727

In-hospital Complications, n (%)			OR	95% CI	SE	*p*
ARDS	0 (0)	0 (0.0)	NA	0 to inf.	NA	NA
Tracheostomy	1.3 (3.3)	1 (2.6)	NA	0 to inf.	NA	NA
Pneumonia	5.6 (14.6)	6.6 (17.2)	1.24	0.3 to 5.0	0.712	0.756
Pleural effusion	0.11 (0.3)	3.3 (8.6)	NA	0 to inf.	NA	NA
Pneumothorax	0.9 (2.3)	1.9 (4.9)	NA	0 to inf.	NA	NA
Hemothorax	1.7 (4.3)	1 (2.6)	NA	0 to inf.	NA	NA
Other complication	5.7 (14.8)	12 (31.2)	0.49	−1.5 to 2.4	0.686	0.148
Mortality	0 (0)	0 (0)	NA	0 to inf.	NA	NA
Discharge location			NA	NA	NA	NA
Home	28.4 (78.5)	23.6 (65.3)				
Rehabilitation clinic	4.2 (11.6)	7.8 (21.6)				
Health care facility	2.8 (7.6)	1 (2.8)				
Other	0.8 (2.2)	3.8 (10.4)				

*Numbers indicate the average of 25 matched imputed sets.

ARDS, acute respiratory distress syndrome; *b*, regression coefficient between clavicle fixation and nonoperative treatment; ICU, intensive care unit; inf., infinite; IQR, interquartile range; NA, no answer (owing to small numbers); OR, odds ratio; RF, rib fixation.

### Secondary Outcomes

Intensive care unit length of stay was comparable between the groups with a median of 3 (1–12) days in the nonoperative group and 2 (1–4) days in the operative group (mean difference, −0.2 days; 95% CI, −2.1 to 1.7; *p* = 0.801). The median DMV was 3 (1–13) days in the nonoperative group versus 7 (2–8) days in the operative group (mean difference, −0.6 days; 95% CI, −2.1 to 0.8; *p* = 0.380). In the nonoperative group, 17.2% developed a pneumonia versus 14.6% in the group, which was treated operatively (*p* = 0.756). There was no in-hospital mortality in either of the groups.

**TABLE 3:** Midterm and Long-term Outcomes After PS Matching of Patients With Combined Clavicle and Rib Fractures

Midterm and Long-term Outcomes	Multiple Rib Fractures and Clavicle Fracture					
	Nonoperative*	Clavicle Fixation*	Regression Coefficient (*b*)	95% CI	SE	*p*
Follow-up 6 wk						
EQ5D-5L index value, mean ± SD	0.77 ± 0.2	0.70 ± 0.2	−0.07	−0.1 to 0.02	0.049	0.135
EQ5D-5L VAS, mean ± SD	71 ± 19	65 ± 19	-5/4	−15.6 to 4.7	5.173	0.292
mMRC, median (IQR)	0 (0–1)	0 (0–1)	0.0	−0.5 to 0.5	0.257	0.989
NRS						
General	2 (1–3)	2 (1–3)	−0.1	−1.1 to 0.9	0.499	0.818
Breathing	0 (0–2)	0 (0–2)	0.0	−0.8 to 0.9	0.418	0.988
Coughing	2 (0–3)	2 (0–3)	−0.1	−1.3 to 1.1	0.599	0.899
Complications, n (%)*			OR	95% CI	SE	*p* value
Pneumonia	0.1 (0.2)	1 (2.4)	NA	NA	NA	NA
Pleural effusion	0.4 (0.9)	1 (2.6)	NA	NA	NA	NA
Pneumothorax	0 (0)	0 (0)	NA	NA	NA	NA
Hemothorax	0 (0)	0 (0)	NA	NA	NA	NA
Follow-up 1 y						
EQ5D-5L index value, mean ± SD	0.84 ± 0.2	0.82 ± 0.2	−0.0	−0.1 to 0.1	0.058	0.658
EQ5D-5L VAS, mean ± SD	75.1 ± 21	81.4 ± 19	6.3	−3.8 to 16.4	5.171	0.226
mMRC, median (IQR)	0 (0–0)	0 (0–0)	−0.0	−0.4 to 0.3	0.206	0.865
NRS (pain)						
General	0 (0–2)	0 (0–2)	−0.3	−1.4 to 0.9	0.573	0.641
Breathing	0 (0–0)	0 (0–0)	−0.0	−0.5 to 0.3	0.206	0.784
Coughing	0 (0–0)	0 (0–0)	−0.0	−0.8 to 0.8	0.413	0.950

Complications			OR	95% CI	SE	*p*
Fracture-related infection clavicle, n (%)	0 (0)	1 (2.6)	NA	NA	NA	NA
Symptomatic clavicle nonunion, n (%)	2.3 (5.9)	0 (0)	NA	NA	NA	NA
Secondary clavicle plate, n (%)	1.6 (4.1)	0 (0)	NA	NA	NA	NA
Persistent pain clavicle, n (%)	0 (0)	1.5 (3.8)	NA	NA	NA	NA
Symptomatic rib nonunion, n (%)	1.2 (1.8)	1 (1.4)	NA	NA	NA	NA
Deceased, n (%)	0.04 (0.1)	0 (0)	NA	NA	NA	NA
Return to work, median (IQR), wk	10 (5–16)	9 (5–15)	1.6	−4.2 to 7.3	2.930	0.591
Return to sports, median (IQR), wk	15 (10–26)	12 (8–26)	−0.0	−7.0 to 6.9	3.585	0.990

*Numbers indicate the average of 25 matched imputed sets.

*b*, regression coefficient between clavicle fixation and nonoperative treatment; IQR, interquartile range; mMRC, Modified Medical Research Council dyspnea scale; OR, odds ratio; NA, no answer (owing to small numbers); VAS, visual analogue scale.

Quality of life was similar between the nonoperative and operative group after 6 weeks (EQ5D-5L, 0.77 ± 0.2 vs. 0.70 ± 0.2; *p* = 0.135) and 1 year (EQ5D-5L, 0.84 ± 0.2 vs. 0.82 ± 0.2; *p* = 0.658) (Table 3). There were no differences in pain between nonoperative and operative treatment after 3 days (median, 3 [2–4] vs. 2 [1–3] days), 5 days (median, 2 [2–3.5] vs. 2 [1–3] days), 7 days (median, 2 [2–3] vs. 0 [1–3] days), 6 weeks (median, 2 [1–3] vs. 2 [1–3] days), and 1 year (median, 0 [0–2] vs. 0 [0–2] days) of follow up. Furthermore, after 1 year follow-up, a total of 8 of 180 nonoperatively treated patients (4%) had a symptomatic nonunion for which 5 underwent secondary clavicle fixation (Supplemental Digital Content, Supplementary Table 1, http://links.lww.com/TA/D11).

## DISCUSSION

It is unclear whether fixation of the clavicle fracture in patients with multiple rib fractures would improve clinical outcome. We hypothesized that clavicula fixation would improve stability of the chest wall and therefore might be beneficial for the patient. Therefore, we performed a multicenter prospective study representing a large cohort of patients with concomitant rib (i.e., median of 6 [interquartile range, 4–6] rib fractures in our cohort) and clavicle fractures to assess whether early clavicula fixation would be beneficial. Clavicle fixation compared with nonoperative treatment of clavicle fractures in patients with combined costoclavicular injuries did not reduce HLOS (median, 6 vs. 9 days; *p* = 0.304). Moreover, clavicle fixation did not reduce intensive care unit length of stay (median, 3 vs. 2 days; *p* = 0.801), pain (day 3, NRS 3 vs. 2, *p* = 0.614; day 5, NRS 2 vs. 2, *p* = 0.780; day 7, NRS 2 vs. 2, *p* = 0.727), or (pulmonary) complications (pneumonia rate, 15% vs. 17%; *p* = 0.756), nor does it improve quality of life (EQ5D at 1 year; mean, 0.84 vs. 0.82; *p* = 0.658).

To date, there are only three small case series that reported on treatment of patients with combined clavicle and rib fractures.^12,13^ Graf et al.^13^ performed a retrospective chart review of patients with blunt chest trauma and concomitant clavicle fractures and compared 36 patients who underwent clavicle fixation to 24 patients who were treated nonoperatively. They found no differences in HLOS and other in-hospital outcomes, which resembles our findings. Our study adds to their results, because we corrected for confounding factors by using PS matching and they did not.^13^ One prospective case series reported on HLOS in 11 patients with combined rib and clavicle fractures following blunt chest trauma.^10^ All patients underwent operative treatment for both injuries during the same session, which resulted in a mean ± SD HLOS of 18.8 ± 8.1 days. This is considerably higher compared with our study (median HLOS, 9 [5–17] days), which could be explained by differences in study population regarding the number of flail chests. However, their study would not be able to assess whether clavicula fixation would improve clinical outcome because of the small sample size and the noncomparative study design. Solberg et al.^12^ reported on a retrospective case series comparing operative and nonoperative treatment in 22 patients with combined rib and clavicle fractures. Nine of 22 patients underwent rib fixation of which 7 were treated with clavicle fixation as well. They reported that operative treatment resulted in lower intensive care unit length of stay and DMV. However, results should be interpreted with caution because the sample size is small and it is unclear whether the reported advantages of fixation should be attributed to rib fixation, clavicle fixation, or a combination of both. So far, no definitive conclusions can be drawn from these studies whether improved outcomes should be attributed to clavicle fixation.

Our study adds to the previous literature, because it represents the only prospective available and largest cohort comparing nonoperative and operative treatment in patients with combined rib and clavicle fractures. In general, our findings do not advocate clavicle fixation in patients with combined costoclavicular injuries. Of course, it does not mean that no patient with combined clavicle and rib fractures could benefit from clavicle fixation. We still believe that some patients might benefit from early clavicle fixation. These include, for instance, multiple injury or geriatric patients who need stability of their shoulder girdle for early mobilization (walking with crutches, wheelchair), paraplegic patients who desperately need their arm function, patients with a floating shoulder, or high-demanding patients (sports, work) who want to benefit from a stable clavicle in the first weeks of rehabilitation. Overall, our findings and considerations can be used as a guideline for patient tailored care and shared decision making when considering treatment options in patients with combined clavicle and rib fractures.

### Limitations

We acknowledge that our study has several limitations. First, although we corrected for many confounders by using PS matching, there remains a possibility for unmeasured confounding as with all observational studies. We believe that we have included all relevant confounders in the PS model, which resulted in adequate comparability regarding these key confounders. Therefore, we expect that the impact of unmeasured confounding is limited. Second, although PS matching enabled us to create comparable groups, it inherently decreased the number of patients available for analysis. The sample size for comparing patients with versus without clavicle fracture fixation was limited and at risk of being underpowered. We have refrained from performing an a priori power analysis because there were no data available to inform this analysis. Nonetheless, our study represents a large cohort of 1.014 patients with thoracic trauma, of which a substantial number of patients (232 [23%]) had concomitant clavicle and rib fractures, which is the largest cohort on this particular group and therefore the best evidence available. Third, the OPVENT study was primarily designed to compare rib fixation with nonoperative treatment of rib fractures, and therefore, patient-reported outcome measures for assessing physical functioning of the upper extremity are not available. Moreover, our primary endpoint, HLOS, could be influenced by multiple organizational factors in the hospital (i.e., waiting time for discharge to a rehabilitation clinic). Part of this study was performed during the COVID-19 pandemic, so it could have influenced HLOS. Although it is uncertain how this might have affected our findings, overall HLOS was not increased for all trauma patients compared with a reference period.^21^ Fourth, the initial patient selection and PS matching have resulted in two comparable groups but with severe thoracic injury (six rib fractures median, 27% radiological flail chest, 44% pulmonary contusion, 48% pneumothorax, 32% hemothorax). Therefore, our results are applicable to this particular group of patients. Fifth, not every clavicle fracture was fixated shortly after admission (64% of patients underwent clavicle fixation within 72 hours and 85% within 1 week). However, we did perform a sensitivity analysis on HLOS from time of operation to discharge, which did not change our results. Lastly, indications for operative treatment of both rib and clavicle fractures were determined by the preferences of the attending surgeons. This may introduce subjectivity to some extent but does, however, resemble daily clinical practice and cultural differences between trauma centers.

## CONCLUSION

We found no evidence that, in patients with combined clavicle and multiple rib fractures, plate fixation of the clavicle reduces HLOS, pain, or (pulmonary) complications, nor that it improves quality of life.

## Supplementary Material
