# Supplementary material for: In patients with combined clavicle and multiple rib fractures, does fracture fixation of the clavicle improve clinical outcomes? A multicenter prospective cohort study of 232 patients
Source: J Trauma Acute Care Surg. 2023 May 11;95(2):249–55. doi: 10.1097/TA.0000000000004001 (PMC10389406; doi:10.1097/TA.0000000000004001)
Supplement: Supplementary file 1 [file jt-95-249-s001.docx]

**Supplementary Table 1**. Multiple regression analysis of patients with combined clavicle and rib fractures

| Outcome variable |  |  | Multiple regression analysis | |  |  |
| --- | --- | --- | --- | --- | --- | --- |
|  | Nonoperative  (n=180) | Clavicle fixation (n=52) | Regression coefficient (b) | 95% CI | SE | *p* value |
| Hospital length of stay | 8 (4-15) | 9 (5-16) | 2.432 | -1.4 to 6.3 | 2.432 | 0.218 |
| Hospital length of stay from clavicle fixation | 5 (1-12) | 5.5 (2.75-9.25) | -1.675 | -6.8 to 3.49 | 2.612 | 0.522 |
| ICU length of stay | 5 (1-12) | 6.5 (2-13) | 0.802 | -1.3 to 1.9 | 0.275 | 0.731 |
| Duration of IMV in days | 5 (2-12) | 5 (2.25-7.75) | -0.160 | -1.4 to 1.1 | 0.632 | 0.800 |
| Duration of epidural analgesia | 5 (4-6.25) | 6 (4.5-8.5) | 1.483 | 0.6 to 2.4 | 0.449 | 0.962 |
| Duration of IV analgesia | 3 (1-6) | 3 (1-6) | 1.267 | -0.2 to 2.5 | 0.694 | 0.104 |
| NRS (Pain) |  |  |  |  |  |  |
| Day 3 | 2 (2-4) | 2 (1-3) | -0.280 | -0.9 to 0.3 | 0.315 | 0.375 |
| Day 5 | 2 (2-4) | 2 (1-3) | -0.023 | -0.6 to 0.5 | 0.282 | 0.933 |
| Day 7 | 2 (2-3) | 2 (1-3) | 0.162 | -0.3 to 0.6 | 0.233 | 0.486 |
| In-hospital complications (n, %) |  |  |  |  |  |  |
| Mortality | 3 (1.7) | 0 (0) | NA | NA | NA | NA |
| Tracheostomy | 7 (3.9) | 1 (1.9) | 0.639 | 0.1 to 7.1 | 1.231 | 0.716 |
| Pneumonia | 37 (20.6) | 8 (15.4) | 1.4 | 0.5 to 3.5 | 0.479 | 0.498 |
| Pleural effusion | 3 (1.7) | 5 (9.6) | NA | NA | NA | NA |
| Pneumothorax | 8 (4.4) | 3 (5.8) | NA | NA | NA | NA |
| Hemothorax | 4 (2.2) | 1 (1.9) | NA | NA | NA | NA |
| Other complication | 47 (26.1) | 16 (30.8) | 0.639 | 0.1 to 7.1 | 1.231 | 0.716 |
| Discharge location |  |  | NA | NA | NA | NA |
| Home | 124 (70.1) | 35 (67.3) |  |  |  |  |
| Rehabilitation clinic | 23 (13) | 11 (21.2) |  |  |  |  |
| Healthcare facility | 21 (11.9) | 1 (1.9) |  |  |  |  |
| Other | 9 (5.1) | 5 (9.6) |  |  |  |  |
| Follow up 1 year |  |  |  |  |  |  |
| EQ5D-5L index value, mean ±SD | 0.82 ±0.2 | 0.82 ±0.2 | -0.024 | -0.1 to 0.1 | 0.039 | 0.534 |
| EQ5D-5L VAS, mean ±SD | 74.31 ±18.9 | 81.7 ±19.4 | 4.982 | -1.9 to 11.8 | 3.486 | 0.153 |
| MMRC, median (IQR) | 0 (0-0) | 0 (0-0) | -0.055 | -0.3 to 0.2 | 0.146 | 0.706 |
| Fractured related infection (n, %) | 0 (0) | 1 (1.9) | NA | NA | NA | NA |
| Symptomatic nonunion clavicle (n, %) | 8 (4.4) | 0 (0) | NA | NA | NA | NA |
| Secondary clavicle plate (n, %) | 5 (2.8) | 0 (0) | NA | NA | NA | NA |
| Persistent pain clavicle (n, %) | 1 (0.6) | 2 (3.8) | NA | NA | NA | NA |

*Numbers indicate the average of 25 matched imputed sets, ICU intensive care unit, IMV invasive mechanical ventilation, IV intravenous, MMRC modified medical research council dyspnea scale NRS numeric rating scale, SD standard deviation, IQR interquartile range, b regression coefficient between clavicle fixation and non-operative treatment, CI confidence interval, OR odd ratio SE standard deviation, NA no answer (due to small numbers)
