# Supplementary material for: In patients with combined clavicle and multiple rib fractures, does fracture fixation of the clavicle improve clinical outcomes? A multicenter prospective cohort study of 232 patients
Source: J Trauma Acute Care Surg. 2023 May 11;95(2):249–55. doi: 10.1097/TA.0000000000004001 (PMC10389406; doi:10.1097/TA.0000000000004001)
Supplement: Supplementary file 2 [file jt-95-249-s002.docx]

**INTRODUCTION**

Thoracic injuries are common, occurring both as isolated chest injuries and in polytrauma patients ^12,14^. In polytrauma patients who sustained blunt chest trauma, the most prevalent fractures include rib (86%), clavicle fractures (19%), or a combination of these injuries (19%) ^4,22^. Both injuries are considered indicators of the severity of thoracic trauma, and may independently increase the risk of mortality ^2,9,13,15,19^. The combination of multiple rib and clavicle fractures could result in decreased stability, compliance, and respiratory support of the chest wall. Therefore, patients with these combined injuries after blunt thorax trauma seem prone to (respiratory) complications and longer hospitalization ^1,16,17^. There is an ongoing debate whether fixation of the clavicle fracture in patients with multiple concomitant rib fractures could be beneficial to provide additional stability to the chest wall and thereby improve clinical outcomes ^22^.

Despite the vast amount of literature describing rib fractures and clavicle fractures as individual entities, the mutual impact and subsequent management of these combined injuries remain unclear. So far, only three studies reported on treatment outcomes in patients with both clavicle and rib fractures.^10,17,20^ Graf et al. reported on a chart review of 60 patients with blunt chest trauma and concomitant clavicle fractures and they did not find any differences between early clavicle fixation versus nonoperative treatment of clavicle fractures^10^. Langenbach et al. reported on 11 patients with flail chest injuries and clavicle fractures, all of whom underwent operative treatment for both fractures and recovered uneventfully ^17^. Solberg et al. presented a retrospective case series of blunt chest trauma patients with combined clavicle and multiple rib fractures, comparing operative (n=9) versus nonoperative (n=7) treatment of the rib fractures ^20^. However, no definitive conclusion could be drawn from these results due to the small number of included patients. In conclusion, the available literature is insufficient to determine whether clavicle fixation improves outcomes in patients with concomitant clavicle and rib fractures.

We, therefore, performed this study with the following research question: does clavicle fixation in patients with combined rib and clavicle fractures affect hospital length of stay, quality of life and other in-hospital outcomes?

**METHODS**

STUDY DESIGN

Data for the current study on combined multiple rib and clavicle fractures was gathered from the database of the OPVENT study. The OPVENT study is an international multicenter prospective cohort study consisting of 1,014 patients with multiple rib fractures and/or flail chest and compared nonoperative treatment to rib fixation. This study was performed in six level 1 trauma centers between January 2018 and March 2021. The study protocol and results on operative versus nonoperative treatment of multiple rib fractures have been published ^3,11^.

In the current study, we focus solely on patients with rib fractures in combination with a clavicle fracture. All consecutive patients of 18 years and older with computerized tomography (CT) confirmed multiple rib fractures (defined as three or more ipsilateral rib fractures) combined with an ipsilateral clavicle fracture were eligible for inclusion. The exclusion criteria were cognitive impairment, non-traumatic rib fractures, and rib fractures due to cardiopulmonary resuscitation. This study adhered to the Strengthening the Reporting of Observational Studies in Epidemiology (STROBE) guidelines (Supplemental Digital Content, <http://links.lww.com/TA/D10>).^7^

STUDY POPULATION

Patient characteristics measured at hospital admission included age, sex, body mass index (BMI), American Society of Anesthesia (ASA) score, presence of chronic obstructive pulmonary disease (COPD), smoking status, trauma mechanism, abbreviated injury scale (AIS) score, injury severity score (ISS), number of fractured ribs, clinical or radiological flail chest (defined as three or more sequential rib fractures in two or more places with (clinical) or without (radiological) paradoxical movement of the chest wall), concomitant injuries (i.e., pulmonary contusion, pneumothorax, hemothorax and, sternum fracture), and laboratory results (specifically pH and base excess).

TREATMENT

Conservative treatment of clavicle fractures consisted of a sling for one week after which shoulder exercises commenced. Fracture displacement of ≥1 shaft width and/or shortening of ≥1 cm were indications for recommending operative treatment ^23^. The decision for operative treatment was made within <72h of admission. Operative treatment of the clavicle was performed with open reduction and internal fixation using plate osteosynthesis. Some patients with displaced clavicle fractures might not have had surgery due to shared-decision making or differences in surgeon preference with regard to operative treatment in some centers. Three of the study hospitals had a preference for operative treatment (Luzerner kantonsspital [LUKS] 24/31 [77%], Elisabeth Tweesteden Ziekenhuis [ETZ] 4/12 [33%] and University Medical Center Groningen [UMCG] 9/37 [24%]), whereas the other three hospitals generally treated patients conservatively (University Medical Center Utrecht [UMCU] 10/98 [11%], Radboud University Medical Center [RUMC] 2/18 [11%], 3/27 [11%]) Haaglanden Medical Center [HMC]). Some patients with combined injuries underwent rib fracture fixation as well. In case of operative treatment of both the clavicle and ribs, these procedures were performed in the same session, The indication for rib fixation was based on clinical and radiological assessment by a trauma surgeon. Indications for rib fixation included flail chest, severely displaced fractures, and chest wall deformity, failure to wean from mechanical ventilation or uncontrolled persistent pain despite maximum administration of analgesia. All rib fractures were treated using locking plates (MatrixRib, Synthes®). The UMCU, LUKS and ETZ performed rib fixation according to protocol in patients with previously mentioned indications. The UMCG, RUMC and HMC did not perform any rib fixations.

PRIMARY AND SECONDARY OUTCOMES

Our primary outcome was to assess whether fixation of a clavicle fracture in patients with combined rib and clavicle fractures affects hospital length of stay (HLOS). Secondary outcomes included intensive care unit length of stay (ILOS), duration of mechanical ventilation (DMV), need for tracheostomy, pneumonia rate and other in-hospital complications, in-hospital mortality rate, and general pain at day 3, 5 and 7 (measured by nurses using a numeric rating scale [NRS]). Mid- and long-term outcomes were measured at the outpatient clinic visit at 6 weeks and using telephone interviews after 12 months. These measures included pain when breathing and coughing (measured using the NRS), quality of life (measured using the EQ5D-5L), dyspnea burden (measured using the modified Medical Research Council [mMRC] dyspnea scale), and return to work and sports in weeks. Other complications included fracture-related infection (defined by the fracture-related infection [FRI] ^18^ and symptomatic clavicle nonunion. Pneumonia was defined as clinical signs and symptoms (two or more present: temperature >38.5°C, auscultation with suspicion for infiltrate or purulent sputum) and/or additional tests (thoracic radiographs with signs of infiltrate, leukocytosis, elevated C-reactive protein) requiring antimicrobial therapy. Acute respiratory distress syndrome (ARDS) was defined according to the Berlin definition ^8^. Symptomatic nonunion was defined as the presence of unsuccessfully healed ribs, confirmed by CT scan, at least 6 months after trauma, with clinical evidence of pain. The EQ-5D-5L is a patient-reported validated questionnaire that measures health-related quality of life based on five dimensions of health: mobility, self-care, usual activities, pain/discomfort and anxiety/depression. The EQ-5D score ranges from 0 to 1, with higher scores indicating better quality of life. In addition, it contains a visual analog scale of 0 to 100 where 0 is the worst health imaginable and 100 the best health imaginable for the patient. The mMRC is a five-category scale that characterizes the level of dyspnea with physical activity, where 0 indicates dyspnea only with strenuous exercise and 4 means already being dyspneic when getting dressed.

ETHICAL APPROVAL

The study was registered in the Netherlands Trial Registry (NTR6833). The institutional review boards of all participating centers approved the study protocol. Informed consent was obtained from all participants

SAMPLE SIZE AND STATISTICAL ANALYSES

All analyses were performed using R statistical software v4.1.2.22. Data were presented as mean with standard deviation (SD), median with interquartile range (IQR) or frequencies and percentages in case of nominal data. The differences in the distribution of the data between study groups was quantified using standardized mean differences (SMD). We performed multiple imputation, creating 25 imputed dataset, to impute missing values for baseline characteristics: AIS head/face/thorax/extremities (4% [39/927]), base excess (29% [266/927]), BMI (3% [25/927]), ISS (4% [39/927]), pH (29% [265/927]), and smoking status (2% [16/927]), using the ‘mice’ algorithm in R. Within each dataset, we performed propensity score (PS) matching to control for confounding. The PS was estimated using binary logistic regression analysis, with clavicle fixation as the dependent variable and age, sex, smoking status, COPD, BMI, ASA score, trauma mechanism, AIS head/face/thorax/abdomen/extremities, ISS, number of fractured ribs, clinical/radiological flail chest, rib fixation and concomitant injuries as covariates in the model. We chose 1:1 nearest neighbor matching, with a caliper of 0.2 of the SD of the natural logarithm of the PS using the MatchIt algorithm in R. After PS matching the baseline characteristics of the two groups were compared and quantified using the Standardized Mean Difference (SMD), where SMD <0.1 indicates adequate comparability between groups. The primary analyses were conducted with the matched cohort. The relationship between clavicle fixation and outcomes was assessed using linear regression analysis for continuous outcomes and binary logistic regression analysis for binary outcomes. Secondary, we performed a multiple regression analysis of all subjects included in the study, with correction for confounding by including potential confounders as covariates in the regression model. A sensitivity analysis was performed on the HLOS, which was measured in this analysis from operation to discharge. Follow-up for the non-operative group started 4.5 days after admission, which was the mean time-to-surgery. This is known as the landmark method to correct for possible immortal time bias.^5^ Analyses were performed separately for each imputed dataset and the results were pooled using Rubin’s rules.

**RESULTS**

PATIENT CHARACTERISTICS

From a total of 1,014 patients, 232 had concomitant clavicle and rib fractures of whom 52 patients (22%) underwent operative treatment for the clavicle fractures and 180 patients (78%) received nonoperative treatment. Follow-up was completed in March 2022 with a completion rate of 85%. The mean time from admission to surgery of the clavicle was 4.5 ± 4.3 days. After propensity score matching on average 39 patients from the operative group were adequately matched to 39 patients from the nonoperative group. All baseline demographic variables before and after propensity score matching are available in Table 1.

PRIMARY OUTCOME

Median HLOS was 6 (3-13) days for the nonoperative group and 9 (5-17) days for the operative group (Table 2). After propensity score matching, no association was found between clavicle fixation and HLOS (mean difference 2.3 days, 95% CI -2.1 to 6.8, p=0.304). Adjustment for confounding through multiple regression analysis yielded similar results (Supplementary Table 1, <http://links.lww.com/TA/D11>).

SECONDARY OUTCOMES

Intensive care unit length of stay was comparable between the groups with a median of 3 (1-12) days in the nonoperative group and 2 (1-4) days in the operative group (mean difference -0.2 days, 95% CI -2.1 to 1.7, *p*=0.801). Median duration of mechanical ventilation was 3 (1-13) days in the nonoperative group versus 7 (2-8) days in the operative group (mean difference -0.6 days, 95% CI -2.1 to 0.8, *p*=0.380). In the nonoperative group, 17.2% developed a pneumonia versus 14.6 % in the group which was treated operatively (*p*=0.756). There was no in-hospital mortality in either of the groups.

Quality of life was similar between the nonoperative and operative group after 6 weeks (EQ5D-5L 0.77 ± 0.2 vs. 0.70 ± 0.2, *p*=0.135) and one year (EQ5D-5L 0.84 ± 0.2 vs. 0.82± 0.2, *p*=0.658). There were no differences in pain between nonoperative and operative treatment after 3 days (median 3 [2-4] vs. 2 [1-3]), 5 days (median 2 [2-3.5] vs. 2 [1-3]), 7 days (median 2 [2-3] vs. 0 [1-3]), 6 weeks (median 2 [1-3] vs. 2 [1-3]) and one year (median 0 [0-2] vs. 0 [0-2]) of follow up. Furthermore, after one year follow up, a total of 8 out 180 (4%) nonoperatively treated patients had a symptomatic nonunion for which 5 underwent secondary clavicle fixation (Supplementary Table 1, <http://links.lww.com/TA/D11>).

**DISCUSSION**

It is unclear whether fixation of the clavicle fracture in patients with multiple rib fractures would improve clinical outcome. We hypothesized that clavicula fixation would improve stability of the chest wall and therefore might be beneficial for the patient. Therefore, we performed a multicenter prospective study representing a large cohort of patients with concomitant rib (i.e. median of 6 (IQR 4-6) rib fractures in our cohort) and clavicle fractures to assess whether early clavicula fixation would be beneficial. Clavicle fixation compared to nonoperative treatment of clavicle fractures in patients with combined costoclavicular injuries did not reduce HLOS (median 6 vs. 9 days, p=0.304). Moreover, clavicle fixation did not reduce intensive care unit length of stay (median 3 vs. 2 days, p=0.801), pain (day 3, NRS 3 vs. 2, p=0.614; day 5 NRS 2 vs. 2, p=0.780; day 7, NRS 2 vs. 2, p=0.727), or (pulmonary) complications (pneumonia rate 15% VS. 17%, p=0.756), nor does it improve quality of life (EQ5D at one year mean 0.84 vs. 0.82, p=0.658).

To date, there are only three small case series that reported on treatment of patients with combined clavicle and rib fractures.^10,17,20^ Graf et al performed a retrospective chart review of patients with blunt chest trauma and concomitant clavicle fractures and compared 36 patients that underwent clavicle fixation to 24 patients who were treated nonoperatively.^10^ They found no differences in HLOS, and other in-hospital outcomes, which resembles our findings. Our study adds to their results, because we corrected for confounding factors by using propensity score matching and they did not.^10^ One prospective case series reported on HLOS in 11 patients with combined rib and clavicle fractures following blunt chest trauma ^16^. All patients underwent operative treatment for both injuries during the same session which resulted in a mean HLOS of 18.8 ± 8.1 days. This is considerably higher compared to our study (median HLOS 9 [5-17] days), which could be explained by differences in study population regarding the number of flail chests. However, their study would not be able to assess whether clavicula fixation would improve clinical outcome due to the small sample size and the noncomparative study design. Solberg et al. reported on a retrospective case series comparing operative and nonoperative treatment in 22 patients with combined rib and clavicle fractures ^21^. Nine out of 22 patients underwent rib fixation of which seven were treated with clavicle fixation as well. They reported that operative treatment resulted in lower ILOS and DMV. However, results should be interpreted with caution as the sample size is small and it is unclear whether the reported advantages of fixation should be attributed to rib fixation, clavicle fixation or a combination of both. So far, no definitive conclusions can be drawn from these studies whether improved outcomes should be attributed to clavicle fixation.

Our study adds to the previous literature, because it represents the only prospective available and largest cohort comparing nonoperative and operative treatment in patients with combined rib- and clavicle fractures. In general, our findings do not advocate clavicle fixation in patients with combined costoclavicular injuries. Of course, it doesn’t mean that no patient with combined clavicle and rib fractures could benefit from clavicle fixation. We still believe that some patients might benefit from early clavicle fixation. These include for instance, polytrauma or geriatric patients who need stability of their shoulder girdle for early mobilization (walking with crutches, wheelchair), paraplegic patients who desperately need their arm function, patients with a floating shoulder, or high-demanding patients (sports, work) who want to benefit from a stable clavicle in the first weeks of rehabilitation. Overall, our findings and considerations can be used as a guideline for patient tailored care and shared decision-making when considering treatment options in patients with combined clavicle and rib fractures.

LIMITATIONS

We acknowledge that our study has several limitations. First, although we corrected for many confounders by using propensity score matching, there remains a possibility for unmeasured confounding as with all observational studies. We believe we have included all relevant confounders in the propensity score model which resulted in adequate comparability regarding these key confounders. Therefore, we expect that the impact of unmeasured confounding is limited. Second, although propensity score matching enabled us to create comparable groups, it inherently decreased the number of patients available for analysis. The sample size for comparing patients with versus without clavicle fracture fixation was limited and at risk of being underpowered. We have refrained from performing an a-priori power analysis as there were no data available to inform this analysis. Nonetheless, our study represents a large cohort of 1.014 patients with thoracic trauma, of which a substantial number of patients (232 [23%]) had concomitant clavicle and rib fractures, which is the largest cohort on this particular group and therefore the best evidence available. Third, the OPVENT study was primarily designed to compare rib fixation to nonoperative treatment of rib fractures and therefore patient-reported outcome measures for assessing physical functioning of the upper extremity are not available. Moreover, our primary endpoint, hospital length of stay, could be influenced by multiple organizational factors in the hospital (i.e. waiting time for discharge to a rehabilitation clinic). Part of this study was performed during the COVID-19 pandemic, it could have influenced HLOS. Although, It is uncertain how this might have affected our findings, overall HLOS was not increased for all trauma patients compared to a reference period^23^. Fourth, the initial patient selection and PS matching has resulted in two comparable groups, but with severe thoracic injury (6 rib fractures median, 27% radiological flail chest, 44% pulmonary contusion, 48% pneumothorax, 32% hemothorax). Therefore, our results are applicable to this particular group of patients. Fifth, not every clavicle fracture was fixated shortly after admission (64% of patients underwent clavicle fixation within 72h and 85% within one week). However, we did perform a sensitivity analysis on HLOS from time of operation to discharge which didn’t change our results. Lastly, indications for operative treatment of both rib and clavicle fractures were determined by the preferences of the attending surgeons. This may introduce subjectivity to some extent, but does however, resemble daily clinical practice and cultural differences between trauma centers.

CONCLUSION

We found no evidence that in patients with combined clavicle and multiple rib fractures, plate fixation of the clavicle reduces hospital length of stay, pain, or (pulmonary) complications, nor that it improves quality of life.

**Supplementary digital content**

**Supplementary Table 1**. Multiple regression analysis of patients with combined clavicle and rib fractures

*Numbers indicate the average of 25 matched imputed sets, ICU intensive care unit, IMV invasive mechanical ventilation, IV intravenous, MMRC modified medical research council dyspnea scale NRS numeric rating scale, SD standard deviation, IQR interquartile range, b regression coefficient between clavicle fixation and non-operative treatment, CI confidence interval, OR odd ratio SE standard deviation, NA no answer (due to small numbers)

Covariates in the model used were: age, ISS, Trauma mechanism, rib fixation, TTSS and AIS head.

**Supplementary Table 2. Strobe checklist**

**REFERENCES**

1. Bakir MS, Langenbach A, Pinther M, Lefering R, Krinner S, Grosso M, et al. The significance of a concomitant clavicle fracture in flail chest patients: incidence, concomitant injuries, and outcome of 12,348 polytraumata from the TraumaRegister DGU®. Eur J Trauma Emerg Surg. 2021 Nov 5;

2. Battle CE, Hutchings H, Evans PA. Risk factors that predict mortality in patients with blunt chest wall trauma: a systematic review and meta-analysis. Injury. 2012 Jan;43(1):8–17.

3. Beks RB, de Jong MB, Sweet A, Peek J, van Wageningen B, Tromp T, et al. Multicentre prospective cohort study of nonoperative versus operative treatment for flail chest and multiple rib fractures after blunt thoracic trauma: study protocol. BMJ Open. 2019 Aug 27;9(8):e023660.

4. Chrysou K, Halat G, Hoksch B, Schmid RA, Kocher GJ. Lessons from a large trauma center: impact of blunt chest trauma in polytrauma patients—still a relevant problem? Scand J Trauma Resusc Emerg Med. 2017 Dec 20;25(1):42.

5. Dekkers OM, Groenwold RHH. When observational studies can give wrong answers: the potential of immortal time bias. Eur J Endocrinol [Internet]. 2021 Jan 1;184(1):E1–E4.

6. Elm E von, Altman DG, Egger M, Pocock SJ, Gøtzsche PC, Vandenbroucke JP. Strengthening the reporting of observational studies in epidemiology (STROBE) statement: guidelines for reporting observational studies. BMJ [Internet]. 2007 Oct 20;335(7624):806–808.

7. Ferguson ND, Fan E, Camporota L, Antonelli M, Anzueto A, Beale R, et al. The Berlin definition of ARDS: an expanded rationale, justification, and supplementary material. Intensive Care Med. 2012 Oct;38(10):1573–82.

8. Flagel BT, Luchette FA, Reed RL, Esposito TJ, Davis KA, Santaniello JM, et al. Half-a-dozen ribs: the breakpoint for mortality. Surgery. 2005 Oct 1;138(4):717–23; discussion 723-5.

9. Graf A, Wendler D, Court T, Talhelm J, Carver T, Beck C, et al. Acute clavicle fixation after blunt chest trauma: effect on pulmonary outcomes and patient disposition. Eur J Orthop Surg Traumatol [Internet]. 2022 Aug 29;

10. Hoepelman RJ, Beeres FrankJP, Beks RB, Sweet AAR, Ijpma FF, Lansink KWW, et al. Non-operative vs. operative treatment for multiple rib fractures after blunt thoracic trauma: a multicenter prospective cohort study. European Journal of Trauma and Emergency Surgery. 2022 Aug 25;1–11.

11. Horst K, Andruszkow H, Weber CD, Pishnamaz M, Herren C, Zhi Q, et al. Thoracic trauma now and then: A 10 year experience from 16,773 severely injured patients. PLoS One. 2017 Oct 19;12(10):e0186712.

12. Horst K, Dienstknecht T, Pfeifer R, Pishnamaz M, Hildebrand F, Pape H-C. Risk stratification by injury distribution in polytrauma patients -- does the clavicular fracture play a role? Patient Saf Surg. 2013 Jul 4;7(1):23.

13. Krug EG, Sharma GK, Lozano R. The global burden of injuries. Am J Public Health. 2000 Apr 10;90(4):523–6.

14. van Laarhoven JJ, Ferree S, Houwert RM, Hietbrink F, Verleisdonk EJM, Leenen LP. Demographics of the injury pattern in severely injured patients with an associated clavicle fracture: a retrospective observational cohort study. World Journal of Emergency Surgery. 2013 Dec 22;8(1):36.

15. Langenbach A, Pinther M, Krinner S, Grupp S, Ekkernkamp A, Hennig FF, et al. Surgical Stabilization of Costoclavicular Injuries - A Combination of Flail Chest Injuries and a Clavicula Fracture. Chirurgia (Bucur). 2017;112(5):595–606.

16. Langenbach A, Pinther M, Krinner S, Grupp S, Ekkernkamp A, Hennig FF, et al. Surgical Stabilization of Costoclavicular Injuries - A Combination of Flail Chest Injuries and a Clavicula Fracture. Chirurgia (Bucur). 112(5):595–606.

17. Metsemakers WJ, Morgenstern M, McNally MA, Moriarty TF, McFadyen I, Scarborough M, et al. Fracture-related infection: A consensus on definition from an international expert group. Injury. 2018 Mar;49(3):505–510.

18. Peek J, Ochen Y, Saillant N, Groenwold RHH, Leenen LPH, Uribe-Leitz T, et al. Traumatic rib fractures: a marker of severe injury. A nationwide study using the National Trauma Data Bank. Trauma Surg Acute Care Open. 2020;5(1):e000441.

19. Solberg BD, Moon CN, Nissim AA, Wilson MT, Margulies DR. Treatment of Chest Wall Implosion Injuries Without Thoracotomy: Technique and Clinical Outcomes. Journal of Trauma: Injury, Infection & Critical Care. 2009 Jul;67(1):8–13.

20. Solberg BD, Moon CN, Nissim AA, Wilson MT, Margulies DR. Treatment of Chest Wall Implosion Injuries Without Thoracotomy: Technique and Clinical Outcomes. Journal of Trauma: Injury, Infection & Critical Care. 2009 Jul;67(1):8–13.

21. Sweet AAR, Beks RB, IJpma FFA, de Jong MB, Beeres FJP, Leenen LPH, et al. Epidemiology of combined clavicle and rib fractures: a systematic review. Eur J Trauma Emerg Surg. 2021 Jun 1;

22. Woltz S, Stegeman SA, Krijnen P, van Dijkman BA, van Thiel TPH, Schep NWL, et al. Plate Fixation Compared with Nonoperative Treatment for Displaced Midshaft Clavicular Fractures. J Bone Joint Surg. 2017 Jan;99(2):106–112.

23. Driessen MLS, Sturms LM, Bloemers FW, Duis HJ ten, Edwards MJR, den Hartog D, et al. The Detrimental Impact of the COVID-19 Pandemic on Major Trauma Outcomes in the Netherlands. Ann Surg [Internet]. 2022 Feb 1;275(2):252–258.

**Figure legend**

**Figure 1.** Flowchart of included patients

**Table legend**

**Table 1.** Baseline characteristics of patients with combined clavicle and rib fracture, before and after propensity score matching

*Numbers indicate the average of 25 matched imputed sets, SMD standardized mean difference, **SMD <0.1 indicates adequate matching, AIS abbreviated injury score, ASA-score American society of anesthesiologists score, BMI Body Mass index, COPD Chronic Obstructive Pulmonary Disease, ISS injury severity score, MVA motor vehicle accident, IQR interquartile range, SD standard deviation, TTSS thoracic trauma severity score

**Table 2.** In hospital outcomes and complications after propensity score matching of patients with combined clavicle and rib fractures

*Numbers indicate the average of 25 matched imputed sets, RF rib fixation, ICU intensive care unit, NRS numeric rating scale, ARDS, acute respiratory distress syndrome, IQR interquartile range, b regression coefficient between rib fixation and non-operative treatment, CI confidence interval, OR odd ratio SE standard deviation, NA no answer (due to small numbers)

**Table 3.** Mid and long term outcomes after propensity score matching of patients with combined clavicle and rib fractures

*Numbers indicate the average of 25 matched imputed sets, MMRC modified medical research council dyspnea scale NRS numeric rating scale, SD standard deviation, IQR interquartile range, b regression coefficient between clavicle fixation and non-operative treatment, CI confidence interval, OR odd ratio SE standard deviation, NA no answer (due to small numbers)
